# Re–Os dating of the Makimine and Shimokawa VMS deposits for new age constraints on ridge subduction beneath Japanese Islands

**DOI:** 10.1038/s41598-024-80799-z

**Published:** 2024-12-03

**Authors:** Tatsuo Nozaki, Yutaro Takaya, Ken Nakayama, Yasuhiro Kato

**Affiliations:** 1 Submarine Resources Research Center, Research Institute for Marine Resources Utilization, Japan Agency for Marine-Earth Science and Technology (JAMSTEC), 2-15 Natsushima-Cho, Yokosuka, 237-0061 Japan; 2 Frontier Research Center for Energy and Resources, School of Engineering, The University of Tokyo, 7-3-1 Hongo, Bunkyo-ku, Tokyo, 113-8656 Japan; 3 Department of Planetology, Graduate School of Science, Kobe University, 1-1 Rokkodai-cho, Nada-ku, Kobe, 657-8501 Japan; 4 Department of Systems Innovation, The University of Tokyo, 7-3-1 Hongo, Bunkyo-ku, Tokyo, 113-8656 Japan; 5 Faculty of Science and Engineering, Waseda University, 3-4-1 Okubo, Shinjuku-ku, Tokyo, 169-8555 Japan; 6 Marine Core Research Institute, Kochi University, B200 Monobe, Nankoku, 783-8502 Japan; 7 Ocean Resources Research Center for Next Generation, Chiba Institute of Technology, 2-17-1 Tsudanuma, Narashino, 275-0016 Japan; 8 Present Address: Faculty of Science and Engineering, Waseda University, 3-4-1 Okubo, Shinjuku-ku, Tokyo, 169-8555 Japan

**Keywords:** Environmental sciences, Ocean sciences, Solid Earth sciences

## Abstract

Ridge subduction is a trigger of thermal metamorphism and hydrothermal activity; thus, it is an important process for understanding geological history of accretionary complexes. However, determining the timing of ridge subduction is often challenging owing to metamorphism and poor microfossil preservation. Some Besshi-type volcanogenic massive sulphide (VMS) deposits associated with in situ greenstone originated by hydrothermal mineralisation on a sediment-covered mid-ocean ridge (MOR); thus, their depositional ages constrain the timing of ridge subduction. Here, we report Re–Os isochron ages of the Makimine and Shimokawa VMS deposits in southwest and northeast Japan. The Re–Os isotope ratios exhibit well-defined linearity and their isochron ages are 89.4 ± 1.2 and 48.2 ± 0.9 Ma. Considering (1) the almost same depositional ages of the VMS deposits with surrounding sedimentary rocks; (2) their close association with in situ greenstone and absence of chert; (3) their radiogenic Pb isotope composition; (4) their high sulphur isotope (δ^34^S) composition with a wide variation; and (5) high thermal gradient in the Makimine area, we inferred the depositional setting of both VMS deposits to be a sediment-covered MOR in a shelf sea. Thus, the VMS deposits were formed just before Izanagi–Pacific Ridge subduction beneath the paleo-Japanese Islands.

## Introduction

**Fig. 1:**
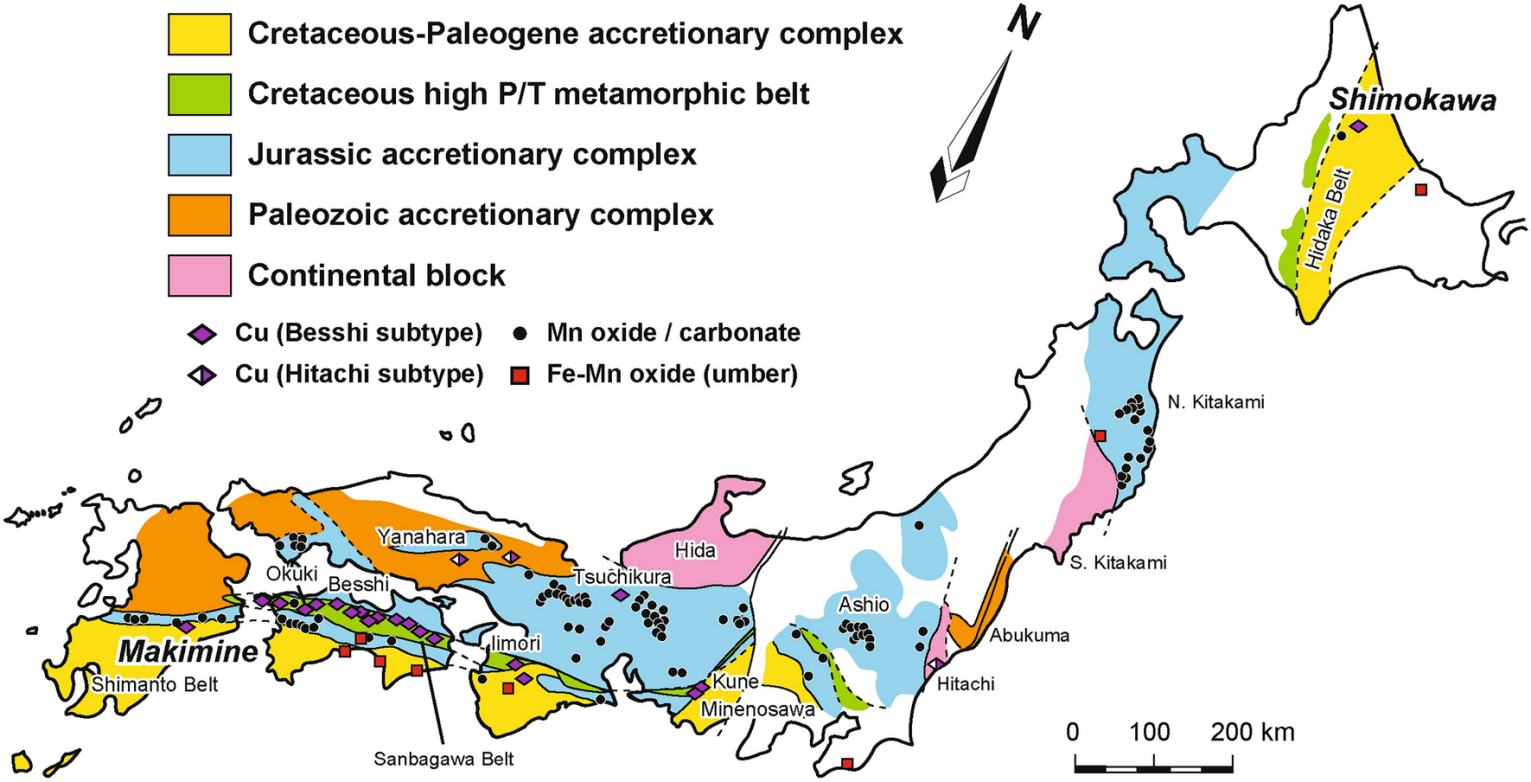
Simplified geotectonic map of the Japanese Islands and locations of representative Besshi-type volcanogenic massive sulphide (subdivided into Besshi and Hitachi subtypes), Mn oxide/carbonate, and Fe–Mn oxide (umber) deposits. Modified from Ref.^4^.

Active continental margins, including the Japanese Islands, include accretionary complexes produced by underplating in the subduction zone^1–3^. Accretionary complexes have an oceanic plate stratigraphy comprising oceanic crust (basalt) and pelagic (chert), hemipelagic (siliceous mudstone), and terrigenous (sandstone and mudstone) sediments in ascending order, and may include mineral deposits such as volcanogenic massive sulphide (VMS), Fe–Mn oxide (umber), and Mn oxide/carbonate deposits formed on the paleo-seafloor^4–6^ (Fig. 1). Two types of VMS deposits occur in the Japanese Islands: (1) Kuroko-type deposits, formed by volcanic activity during the Japan Sea opening in a rifting or back-arc setting^7^; and (2) Besshi-type deposits, formed by volcanic activity on a paleo mid-ocean ridge (MOR)^4,8^.

Besshi-type VMS deposits occur widely in Japan (Fig. 1) but are most densely distributed in the Cretaceous high-P/T metamorphic belt (Sanbagawa Belt), which includes the Besshi deposit type locality in Ehime Prefecture^4^ (Fig. 1). The mineralisation age of the Besshi-type VMS deposits in the Sanbagawa Belt is ca. 150 Ma by Re–Os isotope dating, and because of their close association with hanging-wall quartz schist derived from chert^8,9^, they are considered to have formed on a pelagic MOR^10,11^. Besshi deposits also occur densely in the Shimanto Belt and its northern extension, the Hidaka Belt^4^ (Fig. 1). Several Besshi-type VMS deposits in the Shimanto and Hidaka Belts are associated with in situ greenstone (basalt) and phyllite/sandstone sequences that lack chert. Because in situ greenstone has a geochemical affinity with MOR basalt (MORB) and intruded into the phyllite/sandstone sequences, the MOR must have been located close to the paleo-Japanese Islands^12–14^. Thus, the formation age of Besshi-type VMS deposits provides a new way to constrain the timing of ridge subduction and eruption age of their wallrock (basalt). Here, we report on the tectonic settings, depositional environments, and Re–Os isochron ages of the Makimine and Shimokawa VMS deposits associated with in situ greenstone in Miyazaki and Hokkaido Prefectures, Japan (Fig. 1).

## Geological setting and samples

References^6,15^ have described the geology of the Makimine and Shimokawa VMS deposits. A brief summary follows.

**Fig. 2:**
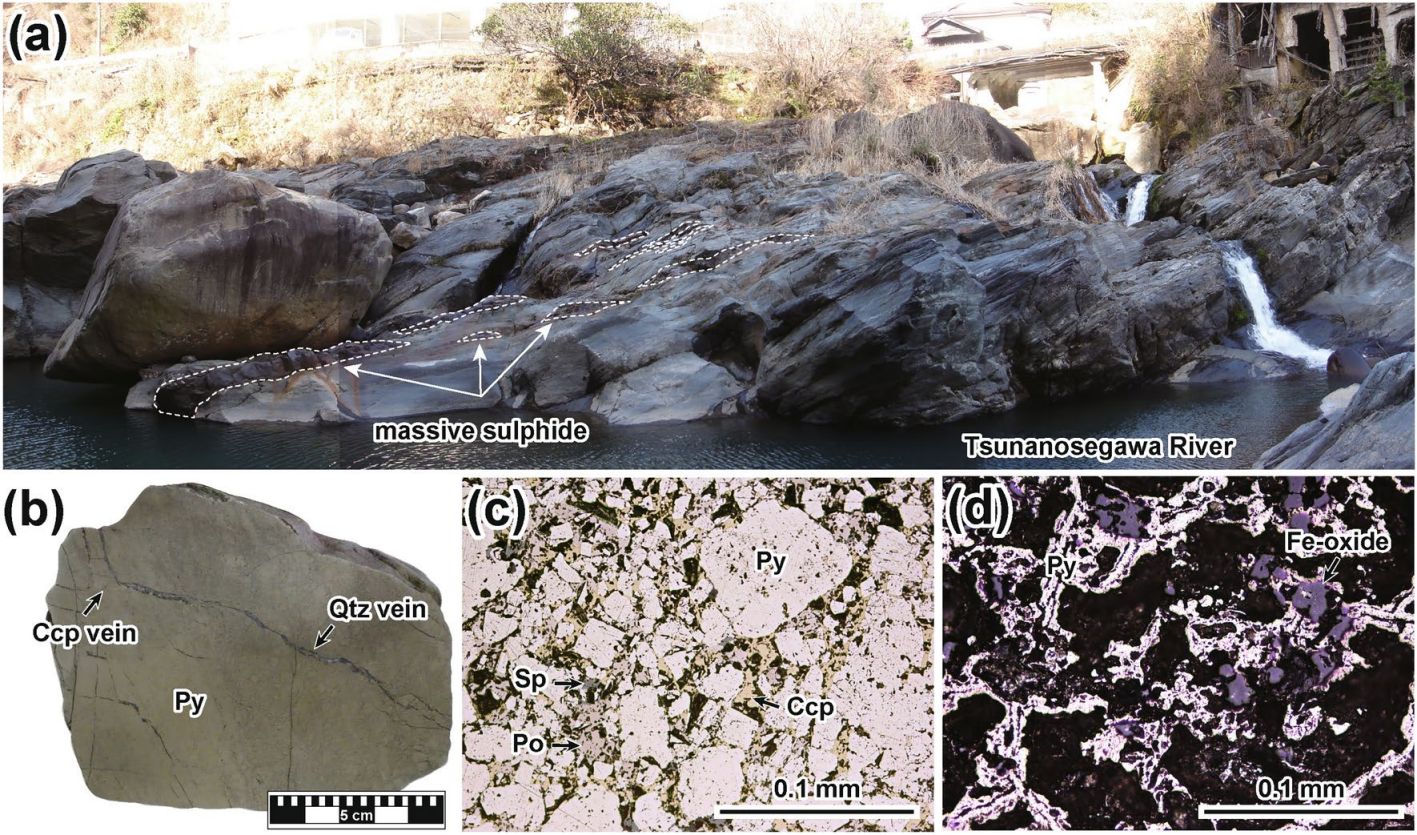
Photographs and microphotographs of the Makimine deposit, modified from Ref.^6^. (**a**) Outcrop photograph: Several massive sulphide layers intercalate phyllite and sandstone. (**b**) Representative hand specimen of massive sulphide ore dominated by pyrite (sample MK11). Microphotographs under reflected light of samples (**c**) MK01 and (**d**) MK23. Sample MK23 was not used in the Re–Os isotope analysis because it contains abundant silicate and oxide minerals. *Ccp* chalcopyrite, *Qtz* quartz, *Po* pyrrhotite, *Py* pyrite, *Sp* sphalerite.

At least 28 Besshi-type VMS deposits of various sizes occur in the Northern Shimanto Belt, Kyushu District, southwest Japan. The Northern Shimanto Belt is the non-metamorphosed or weakly metamorphosed Cretaceous accretionary complex whose maximum degree of the regional metamorphism is up to pumpellyite-actinolite facies^2–4^. The largest is the Makimine VMS deposit (Fig. 1), which was discovered in 1657. It was mined until 1967; total crude ore production was 4.5 Mt, with an average Cu grade of 1.9 wt%. The deposit is hosted in the Makimine Formation, Kamae Subgroup, Northern Shimanto Belt^16^. This formation consists dominantly of phyllite and sandstone, but greenstone (basaltic sill) is often intercalated in the sedimentary rocks (Fig. 2a); radiolarian fossils indicate a formation age of Santonian to middle Campanian (86.3–77.9 Ma)^16,17^. The main constituent sulphide minerals of the massive sulphide are pyrite, pyrrhotite, and chalcopyrite with sphalerite and magnetite. Other minerals are quartz with minor chlorite, sericite, calcite, and garnet. In the Makimine area, greenstone sometimes intruded into phyllite and sandstone, with a chilled margin, and may have included phyllite xenoliths; further, the greenstone has a MORB-like geochemical signature^12,13^. The greenstone is thus considered to be in situ and to have formed on a paleo-MOR covered by terrigenous sediment. In situ greenstone is found in the Northern Shimanto and Hidaka Belts, and its eruption age becomes younger from southwest to northeast Japan^12–14^. Samples of the Makimine sulphide were collected for this study along the Tsunanosegawa River, Nobeoka City, Miyazaki Prefecture (Fig. 2a). Pyrite is the most abundant mineral in the sulphide samples, which also contain pyrrhotite, chalcopyrite, and sphalerite (Fig. 2b–d). The Makimine sulphide can be grouped into two types under the microscope: (1) colloform pyrite-rich one with some pyrrhotite and Fe-oxide mineral (Fig. 2d), and (2) euhedral pyrite-rich one with a matrix of pyrrhotite, chalcopyrite, and sphalerite (Fig. 2c). Twelve sulphide samples were used for the Re–Os isotope analysis (Supplementary Table S1).

The Shimokawa VMS deposit occurs in the Hidaka Belt, Hokkaido Prefecture, northeast Japan (Fig. 1), which consists of Zones S, U, R, and Y from west to east. In situ greenstone occurs in Zones S, U, and R^18,19^, but the largest in situ greenstone, the Shimokawa greenstone block, which hosts the Shimokawa deposit, is in Zone S. This greenstone block is composed mainly of dolerite swarms and intercalated mudstone. The Shimokawa VMS deposit was discovered in 1933 and mined from 1942 to 1982; total crude ore production was 6.85 Mt, with average Cu and Zn grades of 2.34 and 1.0 wt%. The constituent sulphide minerals are mainly pyrite, pyrrhotite, chalcopyrite, sphalerite, and magnetite, with minor pentlandite, mackinawite, cubanite, and galena, and other minerals quartz, calcite, siderite, dolomite, sericite, chlorite, actinolite, epidote, apatite, and talc^15,20^. The Shimokawa deposit comprises the Nakanosawa and Ochiaizawa orebodies. Only the Ochiaizawa orebody has been mined, but the Nakanosawa orebody resource is estimated to be on the same scale as the Ochiaizawa orebody. No microfossils have been reported from the Shimokawa greenstone block, but a zircon U–Pb age (weighted average concordia age of the youngest clusters) of 47.0 ± 11.0 Ma from the Iwaonai flysch, adjacent to the Shimokawa greenstone block on its east side, is interpreted as the sedimentary age of the paleo-seafloor^19^. Ten sulphide samples (two samples from the Ochiaizawa orebody and eight drill core samples from the Nakanosawa orebody were used for the Re–Os isotope analysis (Supplementary Table S2). Our samples can be classified into (1) pyrite-rich ores whose matrices are mainly composed of gangue silicate minerals with some amounts of chalcopyrite, pyrrhotite, and sphalerite as well as very minor galena (Supplementary Fig. S1a,b) and (2) pyrite-rich ores whose matrices are mainly filled with chalcopyrite, pyrrhotite, and sphalerite (Supplementary Fig. S1c,d) (see Ref.^15^ for their detailed petrographic features).

## Analytical methods

Re and Os concentrations and Os isotope ratios were determined by the isotope dilution method after Carius tube digestion^21^. Approximately 50 mg of powdered sample of sulphide mineral aggregate obtained by CH_2_I_2_ heavy liquid separation (i.e., bulk sulphide samples after removing silicate minerals) was spiked with ^185^Re and ^190^Os and digested in 10 mL of inverse aqua regia in a sealed Carius tube at 220 °C for 24 h, followed by solvent extraction of Os using the CCl_4_ solution^22^, back extraction of Os to the HBr solution^23^, purification of Os by microdistillation^24^. From the aqueous phase remaining after the CCl_4_ extraction of Os, Re was separated in an anion exchange resin^25^.

The Re and Os isotope compositions were measured in static multiple Faraday collector mode and pulse-counting electron multiplier mode of a thermal ionisation mass spectrometer (TIMS, Thermo Fisher Scientific TRITON) at JAMSTEC, respectively. For precise analysis of the Re isotope composition, a total evaporation method^26^ was used to eliminate the effect of instrumental mass fractionation during the measurement. The total procedural blank was 7 pg for Re and 2 pg for Os with a ^187^Os/^188^Os ratio of 0.15. Our Re–Os analytical procedures have been fully described elsewhere^5,10,11,27^.

## Results and discussion

**Fig. 3:**
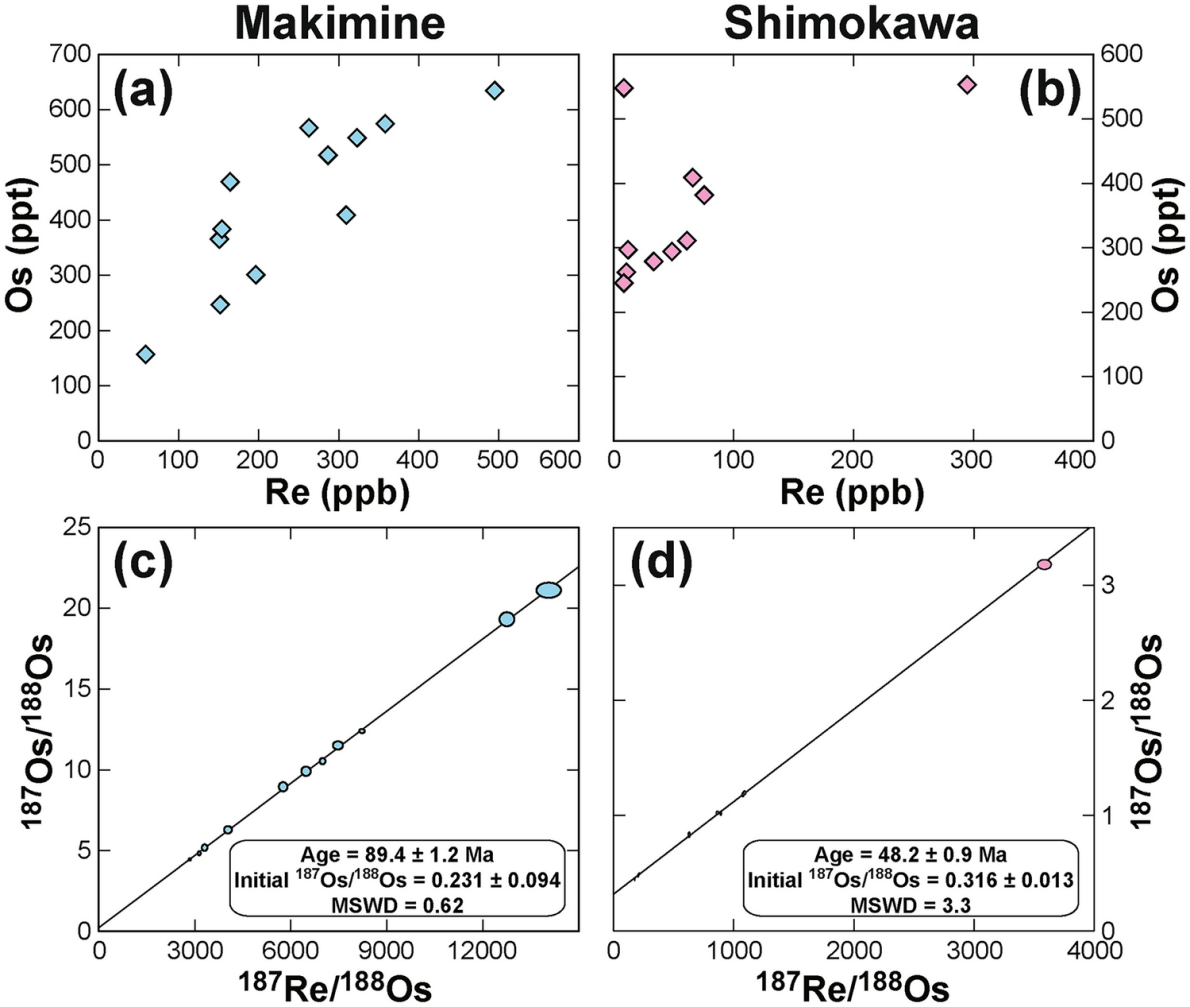
Re–Os concentrations and isotope ratios of the Makimine and Shimokawa deposits and their Re–Os isochrons, drawn with Isoplot v. 4.15 software^43^. MSWD, mean square weighted deviation.

The Re and Os concentrations in the sulphide mineral aggregate of the Makimine deposit were 59.0–495 ppb and 157–635 ppt, respectively, with ^187^Re/^188^Os and ^187^Os/^188^Os ratios of 2600–14,000 and 4.4–21.1, respectively (Fig. 3a,c, and Supplementary Table S1). In the Shimokawa deposit, Re and Os concentrations were 8.8–295 ppb and 246–554 ppt, respectively, and ^187^Re/^188^Os and ^187^Os/^188^Os were 81.4–3590, and 0.45–3.2, respectively (Fig. 3b,d, and Supplementary Table S2). The ratios of ^187^Re/^188^Os and ^187^Os/^188^Os exhibited a well-defined linearity. The Re–Os isochron of the Makimine deposit yielded an age of 89.4 ± 1.2 Ma with an initial ^187^Os/^188^Os ratio (^187^Os/^188^Os_*i*_) of 0.231 ± 0.094 and a mean square weighted deviation (MSWD) of 0.62 (Fig. 3c) when sample MK04, which had large uncertainty due to its low ^187^Os intensity during TIMS measurement, was excluded (Supplementary Table S1). When this sample was included, the Re–Os age was the same, with a similar ^187^Os/^188^Os_*i*_ of 0.232 ± 0.094 and MSWD of 0.57. The Re–Os isochron age of the Shimokawa deposit was 48.2 ± 0.9 Ma, with ^187^Os/^188^Os_*i*_ of 0.316 ± 0.013 and MSWD of 3.3, excluding sample SMK03 (Fig. 3d). The reason this sample deviated from the Re–Os isochron was not clear. The Re–Os isochron determined using all samples yielded an age of 47.5 ± 1.4 Ma, ^187^Os/^188^Os_*i*_ of 0.332 ± 0.024, and MSWD of 34. These Re–Os ages and ^187^Os/^188^Os_*i*_ values are equivalent within their uncertainties, though the deviation of sample SMK03 led to a much larger MSWD.

**Fig. 4:**
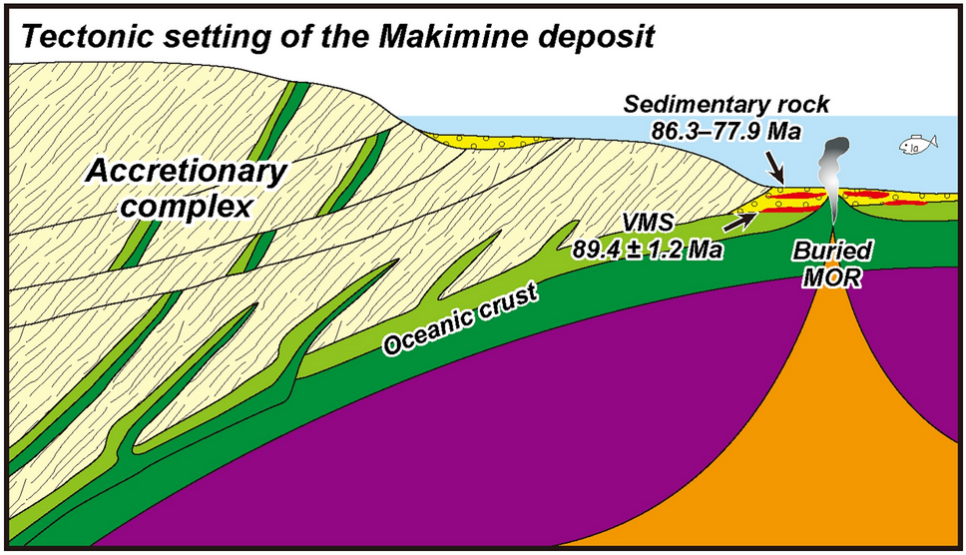
Conceptual diagram of the tectonic setting of the Makimine deposit. A mid-ocean ridge (MOR), located close to the Japanese Islands before its subduction, was covered by terrigenous sediments, which are now observed as phyllite and sandstone on land. Modified from Ref.^6^.

The sedimentary age of the phyllite and sandstone associated with the Makimine deposit was estimated from the included radiolarian fossils to be Santonian to middle Campanian (86.3–77.9 Ma)^16^; this age is about the same or at least 3 million years younger than the Re–Os isochron age of the massive sulphide (89.4 ± 1.2 Ma; Fig. 3c). The occurrence in this area of in situ greenstone with a geochemical composition similar to that of normal MORB (N-MORB) and lacking chert, as well as a short travel time of the oceanic crust from eruption on a paleo-seafloor to accretion in the subduction zone at the northernmost part of the Northern Shimanto Belt including the Makimine VMS deposit estimated by microfossil ages of sedimentary rocks^4,28^, indicate that the massive sulphide was precipitated in a shelf sea supplied with terrigenous sediment from the continental crust. The Pb isotope composition of the Makimine VMS deposit is more radiogenic than that of Pacific MORBs owing to contributions from the surrounding sediment, which had a more radiogenic Pb isotope end-member composition^29^. The S isotope composition (δ^34^S) of the Makimine deposit ranges from + 4‰ to + 7‰, compared with 0‰ to + 3‰ for the Besshi-type VMS deposits^4^ and + 2‰ to + 4‰ of Cu-rich ore of the Besshi VMS deposit^30^ in the Sanbagawa Belt which were similar to the magmatic sulphur isotope composition in footwall rocks leached by hydrothermal fluid through water–rock interaction (δ^34^S = 0‰ ± 3‰)^31,32^ and were precipitated on a pelagic, sediment-barren MOR, whose pelagic depositional setting was endorsed by the close association of chert-origin quartz schist with massive sulphides^4,6,8^ and their Re-Os isochron ages^10,11^. The higher δ^34^S of the Makimine deposit from + 4‰ to + 7‰ has been interpreted due to input of seawater sulphate that was reduced under the relatively closed hydrothermal conditions in the hemipelagic sedimentary pile (sediment-covered ridge such as Middle Valley at the northern end of Juan de Fuca Ridge and Escanaba Trough in southern Gorda Ridge)^4,33^. The metamorphic mineral assemblage occurrences also indicate that the geothermal gradient in the Makimine area is higher than in the circumjacent geological units, which indicates the existence of a heat source beneath the Makimine area^34^. The pyrite grains both in massive sulphide and surrounding phyllite are deformed and oriented to the same direction^6^, suggesting the massive sulphide and phyllite were deformed simultaneously before accretion. Moreover, mm- to cm-sized silicate mineral-rich pebbles and cobbles are often included in massive sulphide^6^. These geological, geochemical, and geochronological lines of evidence strongly suggest that the Makimine deposit was formed on a sediment-covered MOR, replacing unconsolidated sediment (Fig. 2a), before the ridge subducted beneath the paleo-Japanese Islands (Fig. 4). This tectonic setting is consistent with the reconstructed global plate tectonics in the Northwest Pacific during Late Cretaceous to Paleogene time^35,36^. Thus, the Re–Os isotope age of the Makimine deposit shows that VMS deposition on the paleo-seafloor occurred just before subduction of the Izanagi–Pacific Ridge beneath the Japanese Islands (Fig. 4). The similar to slightly younger ages of the phyllite and sandstone can be explained by poor microfossil preservation due to thermal recrystallisation caused by hydrothermal mineralisation at the time corresponding to the Re–Os isochron age.

The depositional setting of the Shimokawa VMS deposit is similar to that of the Makimine VMS deposit. The Re–Os isochron age of 48.2 ± 0.9 Ma is equivalent to the zircon U–Pb age of the sedimentary rock from the Iwaonai flysch (47.0 ± 11.0 Ma; the two youngest concordant ages of 46.4 ± 0.8 and 48.1 ± 0.8 Ma^19^); therefore, the sediment deposition on the paleo-seafloor was contemporaneous with the subseafloor replacement VMS mineralisation^37^. The Pb isotope composition is more radiogenic, with higher ^207^Pb and ^208^Pb than in the Pacific MORB because of the involvement of sedimentary rocks in the hydrothermal mineralisation^4^. The δ^34^S values of the Shimokawa sulphides vary from + 4 to + 12‰, as a result of seawater sulphate input and deposition on a sediment-covered MOR^4^. Therefore, as with the Makimine deposit, the depositional setting of the Shimokawa deposit was most likely a buried MOR in a shelf sea before subduction of the ridge beneath the Japanese Islands (Fig. 4). Granitoids in the Hidaka Belt are grouped by eruption age into three age clusters: 46–45, 40–36, and 19–18 Ma^38–40^. The oldest greenstone cluster, which has an N-MORB-like geochemical signature^41^, is associated with the subduction of the Izanagi–Pacific Ridge. Thus, the Izanagi–Pacific Ridge subduction must have taken place at the Hidaka Belt, Hokkaido Prefecture, northeast Japan in between 48.2 ± 0.9 and 46–45 Ma.

A present distance between the Makimine and Shimokawa VMS deposits is ca. 1600 km and the movement speed of ridge subduction from southwest to northeast Japan can be roughly estimated to be 3.9 cm/year by dividing the present distance by the Re-Os age difference of the two VMS deposits (89.3–48.2 = 41.1 Myr). This speed is generally consistent with the reconstructed plate motion of the Pacific Plate relative to the Eurasia Plate at that time (NNW direction at 23.5 cm/year from 100 to 85 Ma, W direction at 20.2 cm/year from 85 to 74 Ma, NW direction at 10.4 cm/year from 74 to 53 Ma, and N direction at 8.9 cm/year from 53 to 48 Ma)^42^. Throughout geological history of the Japanese Islands, ridge subduction has been an important trigger of batholith formation and its concomitant erosion as well as of the formation of paired high- and low-P/T metamorphic belts in accretionary complexes^2^. The Re–Os isochron age of Besshi-type VMS deposits associated with in situ greenstone can constrain the timing of ridge subduction and is thus a powerful tool for understanding the geological history of active continental margins.

## Conclusions

The Makimine and Shimokawa VMS deposits are associated with in situ greenstone and their depositional ages are almost equivalent to those of the surrounding sedimentary rocks. Both VMS deposits were most likely deposited on a sediment-covered MOR just before subduction of the Izanagi–Pacific Ridge beneath the paleo-Japanese Islands. Thus, Re–Os isochron ages of Besshi-type VMS deposits associated with in situ greenstone provide information on the timing of ridge subduction and the geological history of accretionary complexes in active continental margins.

## Supplementary Information

Supplementary Information 1.

Supplementary Information 2.

Supplementary Information 3.

## Data Availability

All data generated or analysed during this study are included in this published article and supplementary information files.
