# Supplementary material for: Re–Os dating of the Makimine and Shimokawa VMS deposits for new age constraints on ridge subduction beneath Japanese Islands
Source: Sci Rep. 2024 Dec 3;14:30094. doi: 10.1038/s41598-024-80799-z (PMC11615321; doi:10.1038/s41598-024-80799-z)
Supplement: Supplementary file 1 — Supplementary Information 1. [file 41598_2024_80799_MOESM1_ESM.pdf]

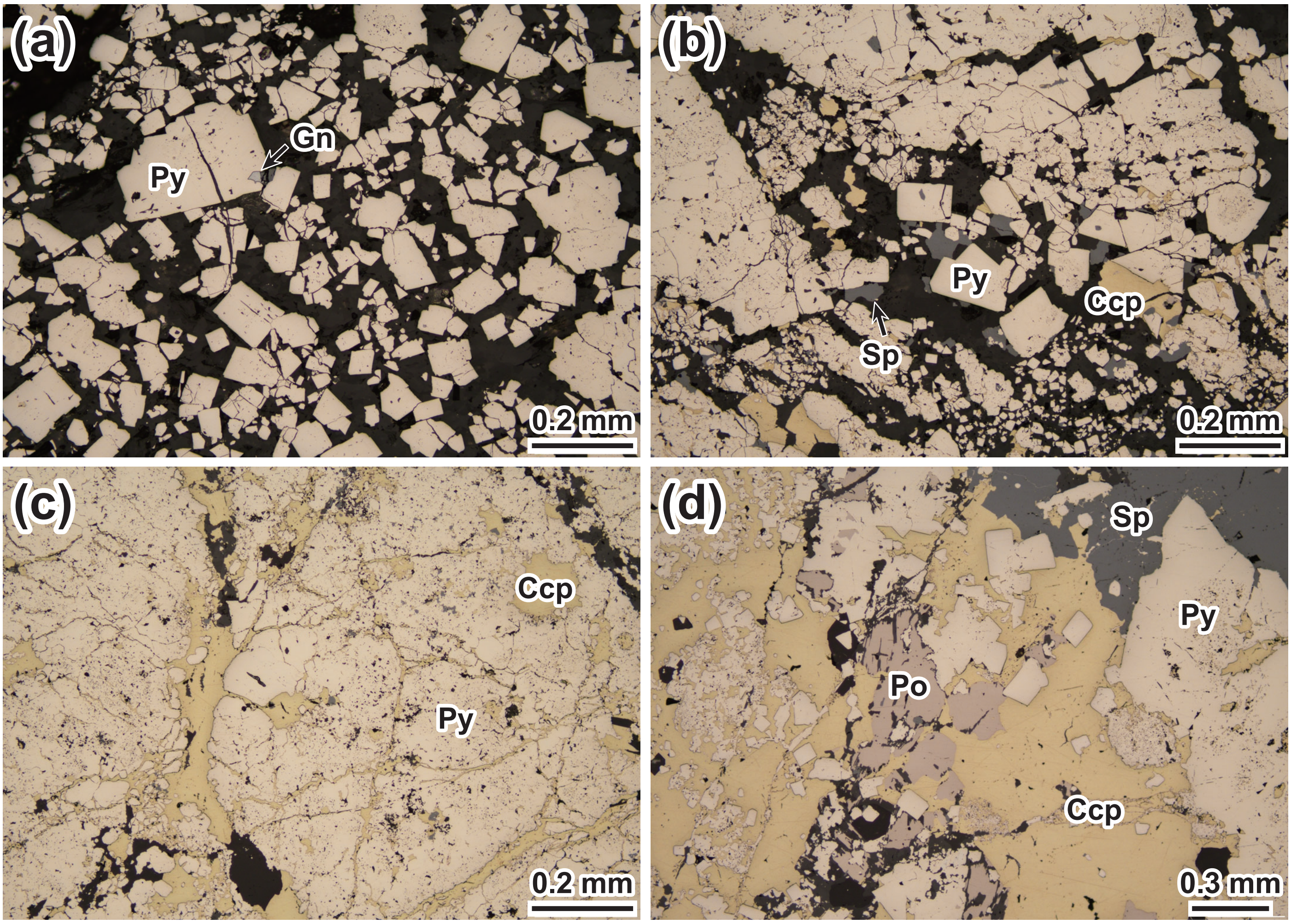

Supplementary Figure S1 Microphotographs of the Shimokawa VMS deposit under reflected light. (a,b) Pyrite-rich samples whose matrices are mainly composed by gangue silicate minerals with some amounts of chalcopyrite, pyrrhotite, and sphalerite as well as very minor galena (sample SMK03 for (a) and SMK10 for (b)). (c,d) Pyrite-rich samples whose matrices are mainly filled with chalcopyrite, pyrrhotite, and sphalerite (sample SMK01 for (c) and SMK02 for (d)). Ccp, chalcopyrite; Gn, galena; Po, pyrrhotite; Py, pyrite; Sp, sphalerite.
